# Quality of life in chronic conditions using patient-reported measures and biomarkers: a DEA analysis in type 1 diabetes

**DOI:** 10.1186/s13561-019-0248-4

**Published:** 2019-11-07

**Authors:** Sixten Borg, Ulf-G. Gerdtham, Katarina Eeg-Olofsson, Bo Palaszewski, Soffia Gudbjörnsdottir

**Affiliations:** 1 Health Economics Unit, Department of Clinical Sciences in Malmö, Lund University, Medicon Village, SE-223 81 Lund, Sweden; 2 Regional Cancer Centre South, Region Skåne, Lund, Sweden; 3 Department of Economics, School of Economics and Management, Box 7082, SE-220 07 Lund, Sweden; 4 Centre for Economic Demography, Lund University, Lund, Sweden; 5 Sahlgrenska Academy, Department of Medicine, University of Gothenburg, SE-413 46 Gothenburg, Sweden; 6 Sahlgrenska University Hospital, Gothenburg, Sweden; 7 Department of Data Management and Analysis, Region Västra Götaland, Lillhagsparken 5, SE-405 44 Gothenburg, Sweden; 8 Centre of Registers Västra Götaland, SE-413 45 Gothenburg, Sweden

**Keywords:** Quality of life, Patient-reported outcomes measures (PROM), Patient-reported experience measures (PREM), Biomarkers, Efficiency analysis, Diabetes, Data envelopment analysis, Benefit-of-the-doubt, C14, I31

## Abstract

**Background:**

A chronic disease impacts a patient’s daily life, with the burden of symptoms and managing the condition, and concerns of progression and disease complications. Such aspects are captured by Patient-Reported Outcomes Measures (PROM), assessments of e.g. wellbeing. Patient-Reported Experience Measures (PREM) assess patients’ experiences of healthcare and address patient preferences. Biomarkers are useful for monitoring disease activity and treatment effect and determining risks of progression and complications, and they provide information on current and future health. Individuals may differ in which among these aspects they consider important. We aimed to develop a measure of quality of life using biomarkers, PROM and PREM, that would provide an unambiguous ranking of individuals, without presuming any specific set of importance weights. We anticipated it would be useful for studying needs and room for improvement, estimating the effects of interventions and comparing alternatives, and for developing healthcare with a broad focus on the individual. We wished to examine if efficiency analysis could be used for this purpose, in an application to individuals with type 1 diabetes.

**Results:**

We used PROM and PREM data linked to registry data on risk factors, in a large sample selected from the National Diabetes Registry in Sweden. Efficiency analysis appears useful for evaluating the situation of individuals with type 1 diabetes. Quality of life was estimated as efficiency, which differed by age. The contribution of different components to quality of life was heterogeneous, and differed by gender, age and duration of diabetes. Observed quality of life shortfall was mainly due to inefficiency, and to some extent due to the level of available inputs.

**Conclusions:**

The efficiency analysis approach can use patient-reported outcomes measures, patient-reported experience measures and comorbidity risk factors to estimate quality of life with a broad focus on the individual, in individuals with type 1 diabetes. The approach enables ranking and comparisons using all these aspects in parallel, and allows each individual to express their own view of which aspects are important to them. The approach can be used for policy regarding interventions on inefficiency as well as healthcare resource allocation, although currently limited to type 1 diabetes.

## Introduction

Noncommunicable chronic diseases, mainly cardiovascular diseases, cancers, chronic respiratory diseases and diabetes, cause 71% of deaths globally, and significant economic burden. Further, modifiable behavioural risk factors increase the risk of these chronic diseases [1–3].

A chronic disease has great impact on a patient’s daily life, with the burden of symptoms and managing the condition, as well as concerns of progression and developing disease complications. Such aspects are captured by Patient-Reported Outcomes Measures (PROM), assessments of wellbeing, abilities and daily life activities, and Patient-Reported Experience Measures (PREM), patients’ experiences of healthcare such as access to services, and the process, e.g. medical encounters and information issues. Adopting a healthy lifestyle may help delaying progression and avoiding both complications and other comorbidities, or treatment may be required to avoid progression. Biomarkers may be useful in monitoring disease activity and treatment effect, and in determining risks of progression and complications. Some risk factors are both prognostic and treatable themselves. Thus, biomarkers provide information on current and future health, PREM address patient preferences regarding healthcare, and PROM describe wellbeing. Which of these are considered important may vary between individuals, and this is also a matter of preferences.

Our general aim was to develop a single measure of quality of life using biomarkers, PROM and PREM together. It would be useful for studying needs and room for improvement, estimating the effects of interventions, and comparing alternatives, as means to develop healthcare with a broad focus on the individual. Developing good practice, identifying providers and clinics that achieve good results, learning from and promoting their practice, are some examples of interventions. Biomarkers, PROM and PREM may be condition specific, thus making the measure useful within the condition only. However, the approach as such should be applicable to chronic diseases overall. With generic data, comparisons across conditions would be possible, enabling priority setting, e.g. allocation of healthcare resources. Furthermore, the quality of life with a condition present could be compared to that of the general population, and the loss due to the condition could be estimated.

There are several methods for measuring quality of life. Health-related quality of life questionnaires can be used together with value sets to estimate indices of quality of life, e.g. the EuroQol (EQ-5D) [4]. Another method is to elicit individuals’ utility functions directly [5]. Yet another option is Willingness to Pay (WTP) studies, which offer different combinations of values that determine quality of life to the respondents, and elicit their WTP for a given set, where a higher WTP signals preference. We perceive difficulties with these methods that we think could be complemented by using efficiency analysis instead [6]. Efficiency analysis also allows individuals to implicitly use their own importance weights of the values used to measure quality of life. Therefore, we wished to examine if efficiency analysis could be used to measure quality of life using PROM, PREM and risk factors, in an application to individuals with type 1 diabetes. Our specific aims were to carry out such an analysis to show that it is feasible, to use the method to study needs and room for improvement, and to outline steps to intervene on causes of poor quality of life. The approach appears useful for evaluating the situation of individuals with type 1 diabetes, in a way that allows ranking based on several values considered in parallel and thereby enables comparisons to be made. The approach can be used for policy regarding interventions on inefficiency as well as healthcare resource allocation, although currently limited to type 1 diabetes.

With our aim to estimate quality of life, using the method of efficiency analysis, we try to join two different fields, and this requires a thorough introduction: Section 2 provides further details on different ways to measure quality of life, details regarding our application to type 1 diabetes and our specific aims, and an overview of efficiency analysis covering the aspects we use in the present work. In Section 3, we present the specific methodology of our approach and our material. The results are presented in Section 4, discussed in Section 5 and we draw conclusions in Section 6.

## Background

### Desired measurement

We aimed to measure quality of life using biomarkers measuring risk factors, PROM and PREM. In our setting, these PROM and PREM are being collected and their data are available for use. Including PROM and PREM should be consistent with measuring quality of life and focus on the individual. Risk factors carry information about risk of future complications and subsequent healthcare costs, but future consequences are associated with uncertainty which may be difficult to judge, and may be valued differently by different patients, and further, not all costs fall upon the individual (externalities). Since a chronic condition impacts the individual as well as the surrounding society, it is relevant to take on both the individual’s perspective as well as a wider perspective, and thus consider risk factors, PROM and PREM simultaneously [7, 8].

Further, we wanted to be able to compare the effects of interventions and to inform choices between alternatives. However, comparisons of several aspects in parallel may give rise to ambiguity, e.g. an individual may be better on one aspect and worse in another. Ideally, we would like a scalar measure that gives an unambiguous ranking of individuals. Furthermore, not all aspects are equally important to everyone, and we wanted our measure to be sensitive to each individual’s own view of what is important. Therefore, we wanted a measure that relies neither on any pre-specified set of weights nor on a set common to everyone, and that avoids ambiguity in comparisons.

### Valuation methods

Given a set of values, or dimensions, a standard approach to measure quality of life is to use a value set for the set of dimensions, to determine the value associated with a combination of values, or health state. There are, for instance, value sets for the commonly used generic questionnaire EQ-5D [4], a frequently used UK value set [9], and a Swedish experience-based value set [10], just to give two examples. Usually, and specifically with the EQ-5D, such valuations are limited to health-related quality of life, and exclude judgments of healthcare involving preferences, such as aspects captured in PREM. Future consequences may be captured by worries about complications, but risk factors as such are usually not included. Thus, typical health-related quality of life studies lack some of the aspects we wished to include.

Furthermore, a value set is designed for a specific questionnaire. For a new set of dimensions, where no value set exists, a new one would have to be developed. In either case, value sets represent the average utility function of the population from which the value set was elicited. The two specifically mentioned sets have used overall valuations of health states to estimate the impact of the different dimensions and levels of the EQ-5D. Even though the individual valuations reflected the individuals’ own preferences, the value sets were derived as the mean impact (i. e. importance) of the involved aspects, and hence does not meet our weight requirement. Using the overall valuation of each respondent would do so, however, but this would entail a significant respondent burden similar to that of developing a value set, infeasible in clinical practice.

Instead of using an existing value set or developing a new one, which relies on a pre-existing utility function which one can tap into to reveal the values of health states, an alternative is to elicit each respondent’s utility functions directly [5]. This would let each individual express their own preferences or importance weights to the dimensions and levels involved and this would meet our weight requirement, but again this procedure would entail additional respondent burden.

Another option is the Willingness to Pay (WTP) study, which offers different combinations of values to the respondents and elicits their WTP for a given set. The attraction of the set depends on the importance of each variable, and its contrast to the respondent’s current level. WTP provides an overall valuation in monetary terms. The individual WTPs reflect each respondent’s own view of the importance of the dimensions involved, thus meeting our requirement on weights. However, it is not straightforward how WTP would be used to estimate the attraction of the respondent’s current state. Freedom from the condition could be offered to the respondents, and the higher their WTP the less attractive their current state (i.e. their experience of having the condition). But if this freedom is unattainable, such as in the case of a chronic condition, it would be an unrealistic offer, and this might affect the WTP estimate. Furthermore, WTP is influenced by the respondent’s budget restriction, and adjustment for this is not entirely straightforward.

Yet another option to measure quality of life of patients is to apply efficiency analysis [11]. Efficiency analysis builds on the notion of a production, that uses one or more types of inputs to produce one or more types of outputs. It does not require any specific set of importance weights, nor does it require the same set of weights for everyone. It also solves the problem of ambiguous comparisons, by comparing individuals with similar weights to each other, and results in a single measure of relative accomplishment, efficiency, assumed to be comparable across different sets of weights. The method is explained in more detail in the Efficiency analysis section. Our use of PROM and PREM relies on data being collected using a straightforward questionnaire, thus causing only a modest respondent burden.

The use of efficiency analysis without any specific set of weights has sometimes been denoted *Benefit of the Doubt* in the literature and uses Data Envelopment Analysis (DEA) to operationalize the efficiency analysis [12]. Hereby it arrives at an individual set of importance weights for each analyzed unit. One example explores the relationship between practices of good governance and quality of life at the municipal level [13], and finds strong as well as weak relationships, i.e. different relative importance. In another study, a composite quality of life index is constructed from eight life domains using a benefit of the doubt-method, with data on the country level [14]. The authors emphasize the need in their setting for allowing different sets of importance weights for different countries, and the undesirability of comparing using a single set of weights everywhere.

Färe and colleagues describe a framework for evaluating healthcare with efficiency analysis [15], which we denote *the capability framework*, consisting of three parts. First, they consider a budget constraint, for the healthcare resources used. Secondly, an intermediate model describes how a healthcare unit produces medical services, using healthcare resource inputs, e.g. physician time, to produce intermediate medical outcomes. The last part is a capability model, that takes the intermediate medical outcomes as inputs and produces patient capabilities, e.g. the patients’ ability to carry out daily activities and the wellbeing of the patient. Thus, they consider two production processes in sequence, which can be optimized under the budget constraint, e.g. to find out how to maximize patient capabilities within the current budget.

Roos and Lundström studied cataract surgery in Sweden, using individual patient data on visual acuity as outputs from the intermediate model, and daily life activities related to the ability to see, such as reading, walking and independent living, which were taken as outputs from the capability model. They looked at changes after surgery, in intermediate outputs and in capabilities, of which the former were the most consistent [16]. Another example of efficiency analysis of healthcare in Sweden is a study of productivity and patient satisfaction in primary care [17].

To the best of our knowledge, efficiency analysis of risk factors, PROM and PREM have not been used simultaneously to measure outcomes in diabetes, despite they all provide relevant information for individuals with diabetes and healthcare decision-makers [7, 8], nor for any other chronic condition. Since efficiency analysis seemed to meet our requirements, namely to unambiguously rank individuals, and to be sensitive to each individual’s view of the importance of the analyzed aspects, we decided to partially adopt the capability framework in our setting, by using efficiency analysis using DEA, on how diabetes care and individuals with diabetes co-produce health in the form of well-controlled risk factors, wellbeing, and favourable experiences of healthcare.

### Application to diabetes

Diabetes Mellitus is a chronic disease with a significant impact on daily life. Individuals with diabetes must be engaged in their disease and its treatment. In Type 1 Diabetes, the ability to produce insulin is lost, and insulin must be injected. The biomarkers glycated hemoglobin level, a measurement of blood glucose values the last 6–8 weeks (HbA1c), systolic blood pressure (SBP), and Low-Density Lipoprotein cholesterol (LDL) are treatable diabetic complication risk factors, and it is important to keep them within recommended intervals. At all times, the individual measures blood-glucose and injects insulin accordingly [18].

Aspects of care and outcomes in diabetes are evaluated in several ways. The levels of HbA1c, SBP, LDL and other risk factors are checked against defined treatment targets [18, 19]. The Swedish National Diabetes Register (NDR) is a quality register that monitors compliance to guidelines, on the individual level [20]. The NDR has developed a diabetes-specific PROM and PREM questionnaire which has been an important way to further improve diabetes care [8, 21, 22], and it is today used in clinical care to collect PROM and PREM in parallel with monitoring risk factors. These risk factors together with PROM and PREM all capture relevant aspects for an individual with diabetes and are available for measuring quality of life as outlined above.

### Efficiency analysis

Efficiency analysis has traditionally been used to analyze production units that transform resources (inputs) into products and services (outputs) [6, 11, 23]. The term technology is used to denote which production of outputs is possible given the level of inputs. The purpose of the efficiency analysis is to determine the actual utility of a unit compared to its potential utility. However, we do not know the limit of what is possible to produce, the frontier, and we do not know how to determine a unit’s true utility, since preferences are usually unknown. Efficiency analysis handles this by taking the best producing units as the frontier, and judgment of a unit’s production is made relative to the frontier.

The correspondence between inputs and outputs is defined by a production function, which determines the maximum output combinations for any given combination of inputs. Outputs are required to be monotone increasing functions of inputs, and preference is assumed to be increasing in outputs and decreasing in inputs. Units that produce as much as possible given their inputs are output efficient, while units producing less are inefficient. A production function is characterized by an assumption of returns to scale, e.g. constant, diminishing or increasing. Efficiency analysis can use parametric production functions, e.g. in a stochastic frontier analysis, as well as non-parametric production functions in DEA. Stochastic frontier analysis is typically used with a single output, or with multiple outputs aggregated into a cost function, provided output prices are available. DEA handles multiple inputs and multiple outputs without the need for prices.

The efficiency analysis estimates each unit’s level of efficiency and hereby indicates units as efficient or inefficient. Each inefficient unit will have a set of peers, efficient units that define the segment on the frontier against which the unit is compared. The unit could learn best practice from its peers and become efficient.

#### Output-oriented efficiency

**Fig. 1:**
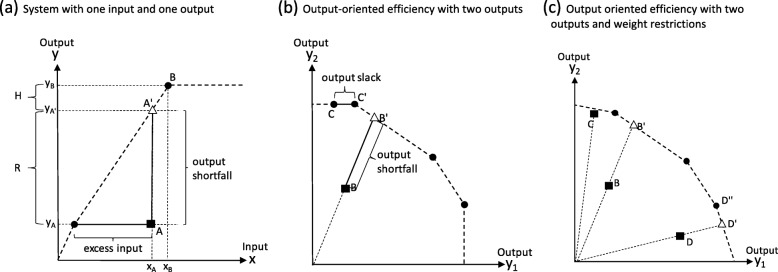
Fronts (dashed lines), efficient units (circles), inefficient units (squares), projected points (triangles), in three systems **a**-**c**

In the case of one input and one output, the ratio of output to input can be used to determine efficiency, and those which produce most in relation to their input define the frontier (circles in Fig. 1 a). Points below the frontier are inefficient (point A), either producing less output than the maximum possible, or using excess input. When there are multiple inputs and multiple outputs, they must all be considered simultaneously.

In an output-oriented system (Fig. 1 b), the output-oriented Farrell efficiency F is the maximum scalar factor with which a unit’s outputs (point B) can be expanded to the front, i.e. along a ray from the origin through the unit’s location to the front (point B′). We take output efficiency as the inverse of F, or the relative distance of the unit compared to the front, also known as the Shephard’s output distance function, D_o_. Farrell efficient units have D_o_ = 1, and inefficient units have D_o_ < 1.

Units can be Farrell efficient but not fully efficient, namely units located on inefficient segments of the frontier (e.g. point C in Fig. 1 b). On such segments, perpendicular to the axes, an output can be increased without decreasing any other output or increasing any input (point C′), however further radial expansion cannot occur without going outside the frontier. The amount of output shortfall along the frontier segment is denoted output slack. However, restricting the weights assigned to outputs to be non-zero can prevent output slack. One approach is the assurance regions method [6, 23], which uses lower and upper limits to the importance of one output relative to another. Inputs can be treated the same way. With such restrictions, frontier segments can no longer be perpendicular to the axes, since all segments must have a slope within the restrictions. The frontier segments adjacent to the axes in Fig. 1 c have slopes identical to the lower and upper limit, respectively. The point C is therefore inefficient (whereas it was efficient with output slack in the system shown in Fig. 1 b). Therefore, when some cases have output slack since unrestricted weighting allows zero weights, we see that after imposing restrictions those cases become inefficient instead.

#### Inefficiency, input and output

When output slack is prevented by the weight restrictions, we subdivide output shortfall into two components. **R** is output shortfall due to inefficiency, i.e. the radial distance to the front, and **H** is the difference in output compared to having another input level than the observed. Figure 1 a illustrates **R** and **H** in an input-output diagram. A unit (point A) can reduce its **R** by becoming more efficient and will produce **y**_**A’**_ if fully efficient. Both efficient and inefficient units may have a positive **H**, which depends on the level of input (**x**_**A**_) allocated to the unit, compared to another unit (e.g. B) that receives more inputs (**x**_**B**_) and therefore produces more output (**y**_**B**_). To reduce **H,** more inputs must be allocated in order to catch up with the other unit.

#### Weights, relative importance and sub-indicator shares

As part of the efficiency analysis, weights are assigned to each of the outputs in a model, for each unit. The ratio of weights between two outputs correspond to the substitution rate between the two outputs and indicate their relative importance.

Sub-indicator shares are a unit’s product of each observed output and its weight and indicate the contribution of each output to total efficiency. They are, unlike the weights, independent of the unit of measurement [12].

## Methods and material

### Evaluating efficiency

**Fig. 2:**
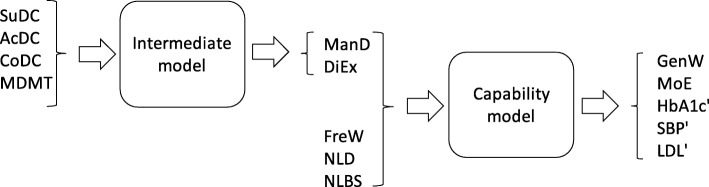
The two production processes in our application of the Capability Approach [15]

In our setting of individuals with diabetes, we adopted the capability framework partially [15]. As we now proceed with our application, we will describe our analysis of *individuals* (instead of analysis of units as in the previous section). Our adaptation consists of two parts: First, our intermediate model describes how healthcare providers and the individual uses judgment of healthcare services as inputs to co-produce intermediate outcomes, namely aspects indicating a patient is successful in managing diabetes and lifestyle factors. Secondly, a capability model takes these intermediate outcomes as inputs and produces patient capabilities and well-controlled risk factors as outputs, thus reflecting the production of wellbeing and low risk of complications. We identified the intermediate PREM outcomes and capabilities and risk factors relevant in our setting (Fig. 2). We focused on the efficiency of the production processes and chose not to adopt the budget constraint part. The efficiency of production, measuring accomplished output relative to an individual’s potential, will provide a measure of an individual’s relative quality of life. The terms efficient and inefficient come naturally in an efficiency analysis, describing how the individuals and their data appear. However, *successful* and *less successful* may be more suitable in the application to diabetes, and we use a combination of these terms.

We used the assurance regions method to control the weights of inputs and outputs [6, 23]. Using type I assurance regions [6], we assumed that the relative importance of different outputs was limited by a value K, such that an output may be no more than K times as important as another output. The same was assumed for the relative importance of different inputs. The restriction applies within each model, and we used the same restriction in both models. With K = 1, all variables are equally important, and with increasing K, the analysis should theoretically converge to the unrestricted analysis. It appears to do so empirically as well.

To choose K, we first considered computational aspects: A very large K allows output slack; we saw non-zero output slack in < 1% of the cases with K = 50. A very small K makes results dependent on small changes in K, e.g. importance weights may be estimated at the lower or upper limits. Preferably, the results should be robust to minor changes of K. Secondly, the motivation for our work builds on the view that different aspects may be important to different individuals. Too small a K violates this view, as it prevents different importance weights, and this should therefore be avoided. We also looked at empirical data on relative importance: value sets for health-related quality of life forms reveal the relative importance of their dimensions, according to the preferences of the respondents used to construct the value set. EQ-5D is a generic instrument frequently used in e.g. economic evaluation [24], with five dimensions: mobility, self-care, usual activities, pain/discomfort and anxiety/depression. We used two value set for EQ-5D, one from respondents valuing hypothetical health states [9], and one from individuals valuing experienced health states [10]. We determined the utility decrement associated with severe problems in each of the five dimensions and computed the ratio of the largest decrement to the smallest. Both value sets resulted in a ratio of 1.7. A generic instrument cannot capture the specific problems of diabetes. We assumed that if we had more dimensions in our analysis e.g. disease specific, we might see a greater ratio. We therefore decided to use this value as a lower limit of K. Finally, we looked at the treatable diabetes comorbidity risk factors HbA1c, LDL and SBP, and their relative importance in predicting all-cause mortality, acute myocardial infarction, stroke and heart failure [25]. They ranked differently depending on outcome, but their relative importance ranged between 3 and 4. Thus empirical data indicate relatively small values although those seen were within PROM, or within risk factors. We therefore used K = 5 in our main analysis, and K = 10 and K = 20 in sensitivity analyses.

### The mix of outputs

We assumed a conceptual model where four factors define an individual’s mix of outputs: the first is the individual’s own characteristics and what is possible for the individual to accomplish, the capability space. Secondly, the individual’s preferences may drive the individual towards showing outputs that are important for the individual, rather than other outputs. The individual’s degree of knowledge of what is important for managing diabetes may affect preferences. Finally, there should be a random component, natural variation but also occasional incorrect questionnaire responses and measurement error in PROM and PREM scores and risk factor levels.

### Material

We used a sample of 1456 individuals with type 1 diabetes, with PROM and PREM data from a questionnaire survey matched with registry data on demographics and risk factors from the NDR [8] (Table 1). They were randomly selected from the NDR using the inclusion criteria of 18–80 years old and at least one HbA1c registered the last 12 months. The survey was conducted in January–November 2015. The questionnaire was developed by NDR to capture aspects considered important by individuals with diabetes [21, 22]. Twelve scales measuring PROM and PREM were developed using Item Response Theory (IRT) [8]. A scale is a translation of response patterns into estimates of an underlying construct such as wellbeing. Scales developed using IRT have several advantages. They allow estimation of latent constructs (e.g. an ability) that cannot be directly observed, they reduce multidimensional sets of items into single estimates of the latent constructs, and these are more robust to missing responses than using the actual responses to the items. Further, they allow items and item response levels to have different difficulties, and assessing changes using the latent constructs can give more accurate estimates of change than using raw test scores [26, 27]. The scales were shown to have acceptable measurement properties, as described in detail elsewhere [8]. The PROM and PREM scales are shown in Table 2. These scales all ranged from 0 (least desirable) to 100 (most desirable).

**Table 1:** Sample characteristics (*n* = 1456)

Patient characteristics	Mean (SD)	Range
Age (years)	49.5 (16.3)	18–80
Male (%)	50%	
Diabetes duration (years)	25.6 (15.9)	0–75
PROM Scales		
GenW General wellbeing	61 (23)	0–100
MoE Mood and energy	66 (22)	0–100
FreW Free of worries	56 (21)	0–100
ManD Manage your diabetes	65 (19)	0–100
DiEx Diet and exercise	57 (23)	0–100
NLD Not limited by diabetes	77 (22)	0–100
NLBS Not limited by blood sugar	71 (27)	0–100
PREM Scales		
SuDC Support from diabetes care	81 (19)	0–100
AcDC Access to diabetes care	70 (20)	0–100
CoDC Continuity in diabetes care	80 (23)	0–100
MDMT Medical devices and medical treatment	78 (20)	0–100
Risk factors		
HbA1c (mmol/mol)	60.9 (11.9)	30–130
Systolic blood pressure (SBP) (mm Hg)	127.4 (14.3)	90–201
LDL-cholesterol (LDL) (mmol/l)	2.40 (0.80)	0.5–8.8

**Table 2:** Patient-Reported Outcomes Measures (PROM) and Patient-Reported Experience Measures (PREM) scales and their abbreviations

PROM scale^a^	Symbol	Scope of items
General wellbeing	GenW	General wellbeing and sleep
Mood and energy	MoE	Depression, difficulty and energy dealing with diabetes
Free of worries	FreW	Concerns about too low or too high blood sugar and complications
Manage your diabetes	ManD	Knowledge, managing diabetes routinely and off routine
Diet and exercise	DiEx	Eating well, staying physically active
Not limited by diabetes	NLD	Barriers for activities, being social
Not limited by blood sugar	NLBS	Blood sugar being too low, too high or unstable
PREM scale^a^	Symbol	Scope of items
Support from diabetes care	SuDC	Support, staff being good listeners
Access to diabetes care	AcDC	Being able to contact and to see physician/nurse
Continuity in diabetes care	CoDC	Being able to see the same physician/nurse
Medical devices and medical treatment	MDMT	Satisfaction with treatment and equipment

^a^See the Material section

We also extracted each individual’s most recent record from the NDR to obtain data on risk factor levels HbA1c, SBP and LDL [8], from at most 1 year before the questionnaire. The risk factors were transformed to satisfy the prerequisites for outputs in an efficiency analysis. We used a linear transformation that ranged from 100, corresponding to the theoretically best risk factor level, down to 0 corresponding to the worst possible risk factor level recordable in the NDR. With this transform we denote the transformed risk factors HbA1c’, SBP’ and LDL’. This enables new samples to be analysed according to the same prerequisites as in the present analysis.

There were only weak, if any, correlations between risk factors and the PROM and PREM scales. Correlations between PROM and PREM scales were in general modest (Additional file 1).

Each individual in the sample was associated with a clinic, and there were 124 clinics with a range of 1 to 73 patients. Sixteen clinics had 30 patients or more.

### Assumptions in DEA

In a DEA efficiency analysis, several assumptions are made. Some were potentially challenged in our setting while others were judged to hold. Two assumptions are made regarding the technology. (1) Free disposability: if a combination of inputs and outputs is possible under the technology, it is also possible to produce less output using the same inputs, or use more inputs to produce the same output (Fig. 1 a) [6]. This assumption should hold in the sense that it is consistent with our view of inefficiency, and it makes it theoretically possible for an inefficient individual to become fully efficient. This may be challenged by our conceptual model of the mix, in case the individual’s outputs already are maximized within the individual’s capability space. On the other hand, then the individual should ideally belong to another front segment which is correspondingly lower. Failure to identify this other front will however lead to overestimating the improvement potential and underestimating the efficiency. (2) Convexity: if two combinations of inputs and outputs are possible, then any mixture of the two is also possible. We judged that this assumption should hold.

Further, three assumptions are made in DEA regarding the inputs and outputs: (3) Preferences are increasing in outputs and decreasing in inputs. All our outputs were desirable and thus satisfy the assumption. The assumption regarding inputs may appear challenged, if an output from one model appears as input in a subsequent model, like in the capability framework. It is preferably high as an output, but acting as input, it is preferable to use no more than necessary. We must keep in mind the roles that a variable plays, depending on which model it appears in. (4) Inputs and outputs are non-negative. This assumption appeared unproblematic and was nonetheless satisfied by the data. (5) There is no noise in the data. Our PROM and PREM scales came from a calculation of scores [8], which contain an element of statistical uncertainty. The risk factors are measurements as well, bound to contain measurement error. However, it is hard to imagine any situation using real word data without any noise in the data, and we judged that the present work would be acceptable as a first study (see discussion). The consequence of breaking (5) will be that the front is not only represented by individuals demonstrating best practice, but also individuals who by chance are very successful when observed, or individuals in both these categories. This ought to translate into a random error in the efficiency measure, probably towards underestimating efficiency.

Further, we made two assumptions in our specific setting: (6) the restrictions on the relative importance of outputs and inputs. With restrictions on the weights, we may use a reference point outside the capability space, in which case we will underestimate the individual’s efficiency. In Fig. 1 c, point D and its projection onto the front D’ illustrates this. In case point D” represent the maximum achievable level of y_1_, then D’ is outside the capability space. Since D is compared to D’, we underestimate its efficiency. Secondly, (7) our conceptual model of the output mix, namely that the mix of output produced is determined by the individual’s capability space, preferences, knowledge and a random component. Empirical data may be required to test this assumption. Whether the model applies both before and after attempts to improve efficiency cannot however be tested with our current data (see discussion).

Thus, to summarize, we may have to expect underestimated efficiency, depending on the nature of the capability space and due to noise in the data, and in any case, we need to expect some random error in our efficiency measurement.

### Production models

We took the perspective of an individual with diabetes, so the healthcare services with support, access, continuity and medical treatment as judged by the individual in the PREM scales SuDC, AcDC, CoDC and MDMT were considered as inputs into the intermediate model, and an individual’s abilities to manage diabetes and lifestyle factors, ManD and DiEx, were considered its outputs.

Subsequently, ManD and DiEx became inputs to the capability model, and we selected the two most general PROM scales, GenW and MoE, and the transformed risk factors as its outputs. Since freedom from worries and limitations (i. e. FreW, NLD and NLBS) appears a necessary requirement for wellbeing (GenW and MoE), we took these as additional inputs rather than capabilities themselves (See discussion).

Hereby we formulated production to take place in sequence in an Intermediate Model (IM), followed by a Capability Model (CM); Fig. 2. We chose decreasing returns to scale.

(ManD, DiEx) = IM(SuDC, AcDC, CoDC, MDMT).

(GenW, MoE, HbA1c’, SBP’, LDL’) = CM(ManD, DiEx, FreW, NLD, NLBS).

The intermediate efficiency describes how well diabetes care and the individual co-produce the individual’s abilities to manage diabetes and lifestyle factors. The capability efficiency describes how well the individual uses his or her abilities to manage diabetes and lifestyle factors and freedom from worries and limitations, to create capabilities in terms of wellbeing and well-controlled risk factors, i.e. to maximize his or her quality of life, at least in terms of the values that we measure.

### Analyses and presentation of results

We estimated the efficiency in each of the production models. Efficiency and output were illustrated by plotting average output in bar charts by quartile group, split by quartiles (Q_i_, i = 1,2,3) of efficiency; group 1: 0 ≤ Do < Q_1_; group 2: Q_1_ ≤ Do < Q_2_; group 3: Q_2_ ≤ Do < Q_3_; group 4: Q_3_ ≤ Do ≤1).

Given known gender differences in capabilities, e.g. men report higher GenW and MoE than women [8], men and women were compared, as were age and duration quartile groups. Kruskal-Wallis tests were used to test for differences in inputs, outputs and efficiency between groups. In order to avoid mass-significance, a *p*-value < 0.001 was used to flag associations. We did not stratify the efficiency analysis by demographic and duration groups as we wished to be able to study differences in efficiency between them.

To determine the contribution of risk factors, and abilities, respectively in the capability model, we present the sub-indicator shares of the capability efficiency. Kruskal-Wallis tests were used to detect group differences.

Out of the diabetes clinics with at least 30 individuals each, five were used in a what-if analysis on the clinic level, where we looked at shortfall due to inefficiency (R), shortfall due to different levels of inputs (H), and estimated output if the individuals at the clinics had been fully efficient and had the same input levels as those belonging to a reference clinic. The purpose was to study the impact of intervening on inefficiency versus reallocating inputs. We selected the clinics with the lowest and highest mean intermediate efficiency, and the three clinics with highest input levels (one clinic was the highest on two inputs). The reference clinic was chosen as one of these such that it would give all the other selected clinics additional output had they received the same input levels.

We performed sensitivity analyses with K = 10 and K = 20 to examine the impact of K.

The analyses were carried out using the *R* software [28], and the *Benchmarking* package [29].

## Results

### Efficiency

**Fig. 3:**
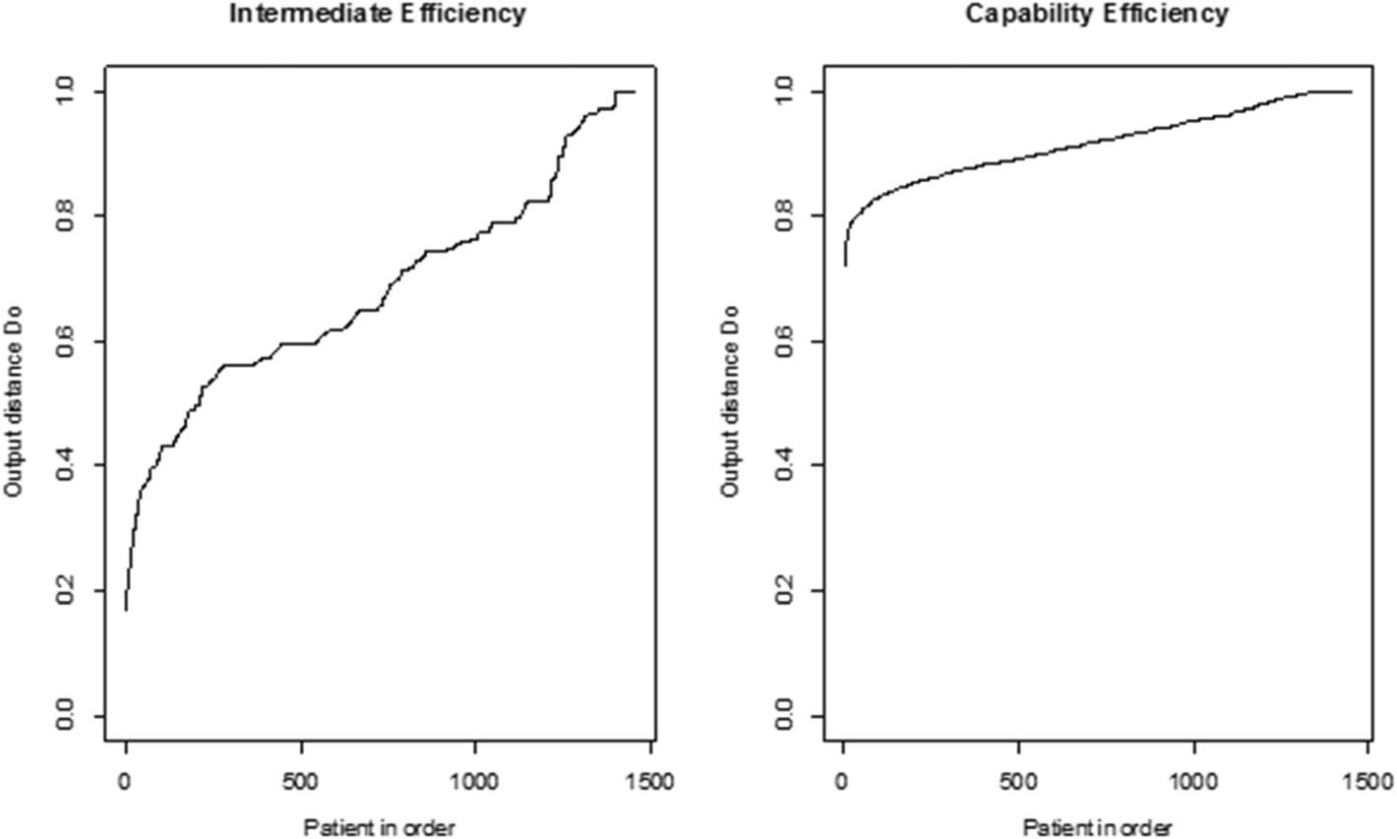
Efficiency in the intermediate and capability models

Around 4% of the individuals were on the frontier in the intermediate model; around 9% in the capability model (Table 3), thus efficiency discriminates between most individuals. Figure 3 shows the distribution of efficiency in the models. The curve was smoother, and the efficiency range was narrower in the capability model (0.7 to 1.0 vs. 0.2 to 1.0) due to the distribution on the risk factors levels. However, judgment of efficiency is made within a model only, so intermediate production is not necessarily worse than capability production.

**Table 3:** Efficiency (proportion on the front, mean efficiency and 95% confidence interval), input and output weights for the intermediate and capability models

(a) Intermediate model											
	Input weights^a^				Efficiency	Output weights^a^					
On the front	SuDC	AcDC	CoDC	MDMT	Mean (95% CI)	ManD	DiEx				
3.8%	0.18	0.26	0.36	0.20	0.68 (0.35; 1.00)	0.65	0.35				
(b) Capability Model											
	Input weights^a^					Efficiency	Output weights^a^				
On the front	FreW	ManD	DiEx	NLD	NLBS	Mean (95% CI)	GenW	MoE	HbA1c’	SBP’	LDL’
8.5%	0.16	0.27	0.28	0.10	0.19	0.92 (0.80, 1.00)	0.12	0.14	0.26	0.24	0.25

^a^Mean weights, normed to unit sum. ‘= transformed. *CI* confidence interval

**Fig. 4:**
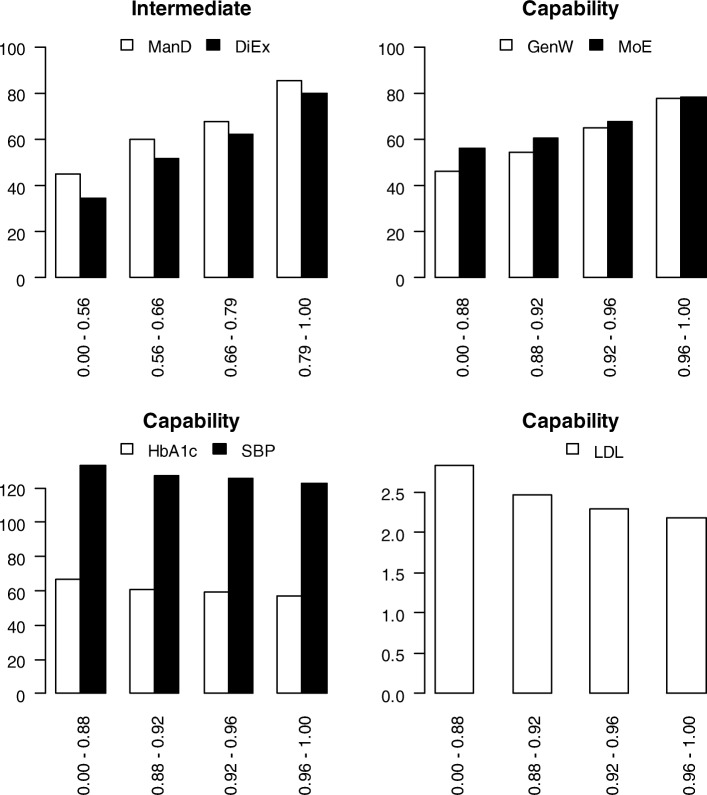
Output by efficiency quartile group in the intermediate and capability models

In both models, there were trends in the outputs consistent with efficient production (Fig. 4), i.e. higher output among more efficient (successful) individuals. The risk factors levels (shown on their original scales on which a lower value is preferable to a higher value) were lower among more efficient (successful) individuals (Fig. 4).

HbA1c had the highest share, 27%, of capability efficiency, followed by SBP and LDL, MoE and GenW (Table 4). The shares of GenW and MoE increase with efficiency whereas the shares of the risk factors decrease (Table 4). The SDs indicate the shares are heterogeneous. Stratifying the individuals by shares being below or and above median, the individuals in the strata with the highest efficiency (12%) had PROM shares above median and risk factor share below. 11% had PROM shares below median, HbA1c and SBP shares above, and LDL below, and among the lowest efficiencies. Several fractions displaying other share patterns were spread across the whole range of efficiency.

**Table 4:** Capability efficiency and its component shares overall and by efficiency quartile group

	All		1st < 0.88		2nd 0.88–0.92		3rd 0.92–0.96		4th ≥0.96	
	Mean	SD	Mean	SD	Mean	SD	Mean	SD	Mean	SD
Capability efficiency	0.92	0.06	0.84	0.01	0.90	0.01	0.94	0.01	0.99	0.03
GenW share (%)	10.7	10.7	6.7	12.0	8.4	14.5	13.4	7.0	14.2	4.2
MoE share (%)	15.9	23.2	7.4	30.3	7.1	25.9	17.5	8.5	31.6	9.4
HbA1c’ share (%)	26.9	22.5	33.4	21.1	31.7	22.7	24.8	21.8	17.6	21.0
SBP’ share (%)	20.7	18.4	25.3	19.1	24.0	17.9	18.1	17.9	15.4	16.9
LDL’ share (%)	25.8	22.4	27.2	22.8	28.8	24.5	26.2	21.3	21.1	20.0
n	1456		364		364		364		364	

#### Subgroups

IRT scores, risk factors and efficiencies are summarized by gender and age group in Table 5, and by duration group in Table 6. Their roles as production inputs and outputs are shown in Fig. 2.

**Table 5:** Scales, risk factors and efficiencies, overall and by gender and age group. Each component’s share of total capability efficiency

	All		Male		Female			18–35 years		36–50 years		51–62 years		63–80 years		
	Mean	SD	Mean	SD	Mean	SD		Mean	SD	Mean	SD	Mean	SD	Mean	SD	
Scales																
GenW	61	23	64	23	58	23	*	61	24	58	24	60	23	64	22	
MoE	66	22	70	21	62	23	*	61	23	64	22	66	22	71	22	§
FreW	56	21	60	20	52	21	*	52	23	56	21	57	21	60	20	§
ManD	65	19	66	19	63	19		64	21	62	19	64	17	69	16	§
DiEx	57	23	58	24	56	23		56	26	53	23	57	22	63	21	§
NLD	77	22	78	22	76	21		77	22	78	21	77	22	76	22	
NLBS	71	27	74	25	68	28	*	70	28	71	27	71	26	70	25	
SuDC	81	19	82	18	79	20	*	80	19	78	20	80	19	84	17	§
AcDC	70	20	71	19	68	21		67	21	68	20	69	20	74	19	§
CoDC	80	23	78	24	82	22	*	80	23	79	23	78	24	80	24	
MDMT	78	20	77	21	78	20		75	21	75	20	79	20	83	18	§
Risk factors																
HbA1c	60.9	11.9	60.3	11.5	61.5	12.2		59.6	13.4	61.4	11.6	61.7	11.8	60.8	10.5	
SBP	127.4	14.3	129.1	13.8	125.7	14.6	*	119.1	11.2	124.8	12.6	130.3	13.2	134.7	14.8	§
LDL	2.44	0.76	2.44	0.75	2.44	0.78		2.53	0.74	2.54	0.80	2.44	0.75	2.28	0.73	§
Efficiency																
Intermediate	0.68	0.17	0.69	0.18	0.67	0.17		0.68	0.19	0.65	0.17	0.67	0.17	0.72	0.15	§
Capability	0.92	0.06	0.92	0.06	0.91	0.06		**0.93**	0.06	**0.91**	0.06	**0.91**	0.06	**0.92**	0.05	§
GenW share (%)	10.7	10.7	11.8	12.2	9.6	8.9		10.3	9.9	10.5	11.6	11.0	11.8	10.8	9.6	
MoE share (%)	15.9	23.2	19.1	25.1	12.7	20.6	*	**11.6**	19.7	**15.2**	22.5	**17.2**	24.5	**19.3**	24.9	§
HbA1c’ share (%)	26.9	22.5	26.2	22.6	27.6	22.5		27.9	22.5	26.4	20.9	27.4	23.7	25.9	23.0	
SBP’ share (%)	20.7	18.4	17.9	17.4	23.5	19.0	*	**29.5**	18.6	**24.3**	18.1	**17.0**	17.0	**1.28**	15.4	§
LDL’ share (%)	25.8	22.4	25.0	22.4	26.6	22.3		**20.7**	20.3	**23.7**	20.6	**27.4**	22.8	**31.1**	24.0	§
n	1456		723		733			351		366		348		391		

* = differences between genders (*p* < 0.001) Kruskal-Wallis. § = difference between age groups (*p* < 0.001). *SD* Standard deviation

**Table 6:** Scales, risk factors and efficiencies, overall and by diabetes duration group

	All		0–12 years		13–23 years		24–36 years		37–75 years		
	Mean	SD	Mean	SD	Mean	SD	Mean	SD	Mean	SD	
Scales											
GenW	61	23	60	23	62	23	61	24	60	22	
MoE	66	22	64	23	65	22	66	22	68	22	
FreW	56	21	53	22	56	21	57	21	60	21	
ManD	65	19	63	18	64	20	64	19	68	17	
DiEx	57	23	57	24	56	24	57	24	60	22	
NLD	77	22	77	21	77	22	78	22	77	22	
NLBS	71	27	72	27	69	28	70	26	71	26	
SuDC	81	19	81	19	81	19	81	18	79	19	
AcDC	70	20	69	21	70	19	70	20	69	20	
CoDC	80	23	79	24	78	23	82	23	79	23	
MDMT	78	20	76	21	77	21	79	19	79	19	
Risk factors											
HbA1c	60.9	11.9	58.9	13.0	62.0	12.4	61.7	11.0	61.0	10.7	¤
SBP	127.4	14.3	124.0	12.6	124.9	14.3	128.1	13.9	132.3	14.6	¤
LDL	2.44	0.76	2.56	0.79	2.46	0.69	2.42	0.74	2.34	0.81	¤
Efficiency											
Intermediate	0.68	0.17	0.67	0.18	0.67	0.18	0.68	0.17	0.71	0.16	
Capability	0.92	0.06	0.92	0.06	0.92	0.06	0.92	0.06	0.91	0.06	
GenW share (%)	10.7	10.7	10.3	10.5	11.6	12.6	10.9	10.7	9.8	9.0	
MoE share (%)	15.9	23.2	13.9	22.0	15.7	23.3	16.3	23.5	17.8	23.9	
HbA1c’ share	26.9	22.5	30.6	23.9	24.3	20.9	25.1	21.9	27.2	22.7	
SBP’ share	20.7	18.4	**23.9**	18.5	**23.8**	18.7	**19.7**	18.5	**15.6**	16.4	¤
LDL’ share	25.8	22.4	**21.2**	20.4	**24.5**	22.1	**28.0**	23.3	**29.6**	22.8	¤
n	1456		353		365		352		376		

* = differences between genders (*p* < 0.001). ¤ = difference between duration groups (*p* < 0.001). *SD* Standard deviation

Intermediate production of ManD and DiEx, using the inputs SuDC, AcDC, CoDC and MDMT, did not differ in efficiency between men and women. Among the inputs, SuDC was higher in men than in women, but CoDC is on the other hand lower, and there were no differences in outputs (Table 5).

The capability production efficiency did not differ between men and women. The inputs FreW and NLBS were higher in men, as were the outputs GenW and MoE, but the output SBP (transformed) was higher in women, i.e. SBP itself is lower.

The shares of MoE and SBP of capability efficiency differed, namely MoE higher in men and SBP higher in women.

The intermediate production in the four age groups was affected both by differences in inputs and efficiency. AcDC and MDMT increased with age, and SuDC was highest among the oldest individuals, and lowest in 36–50 years, and the same pattern was seen in both ManD and DiEx, as well as in intermediate efficiency.

The capability model inputs differed in the four age groups; FreW increased with age, and ManD and DiEx varied with age as mentioned above. Among the outputs, MoE increased with age, LDL was lower above 51 years of age, and SBP increased with age. Thus, production of MoE and LDL was best among older individuals, while production of SBP was better among younger individuals. Efficiency was highest < 35 and > 63 years. MoE and LDL had shares of capability efficiency that increased with age whereas the SBP share decreased with age.

Between duration groups, there were no differences in IRT scores, nor efficiency in any of the production models. However, the risk factors differed between duration groups (Table 6). The shares of SBP and LDL differed by duration, LDL’s share increased and SBP’s share decreased.

### What-if analysis

**Fig. 5:**
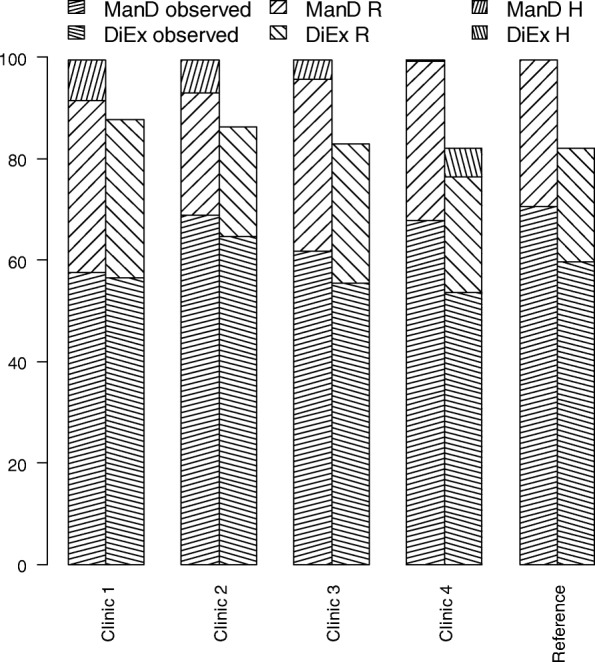
Mean outputs and output shortfall at five diabetes clinics, and a What-If-analysis if clinics 1–4 had the same input levels as the reference clinic

The selected clinics for the what-if analysis are presented in Fig. 5. Clinic 1 was the on average least efficient clinic. The observed output was on average the lowest. Clinic 2 is the most efficient clinic, and even though it has less inputs than the reference clinic, its observed mean outputs were similar to those of the reference clinic (ManD was almost as high and DiEx was higher).

Shortfall due to inefficiency (R) was seen at all clinics, including the reference clinic and clinics 3–4, the latter three selected for having high inputs. Regarding shortfall due to lower inputs (H), shortfall on ManD was seen in Clinics 1–3 and shortfall in DiEx was seen in clinic 4. No such shortfall was seen at the reference clinic (since H is shortfall relative to the reference clinic). At clinics 1–4, for both ManD and DiEx, output shortfall was mainly due to inefficiency (R is greater than H).

If all individuals were efficient (successful), there would still be shortfall in ManD due to lower inputs in clinics 1–3, and allocation of additional inputs would be required to fully eliminate the shortfall. The situation was the same for DiEx at clinic 4. None of the clinics would achieve 100 units of mean DiEx if they were fully efficient. The allocation of inputs appeared insufficient for maximum production of DiEx, even at the reference clinic.

### Sensitivity to restrictions

We used K = 10 and K = 20 in two sensitivity analyses (Additional file 1: Table S2 and Figure S1). Compared to the main analysis using K = 5, the proportion on the front in the intermediate model increases slightly with K, and input and output weights change slightly. In the capability model, the proportion on the front increases slightly more, and input and output weights change slightly. The relative importance analysis was very stable over the choices of K (data not shown).

## Discussion

We used efficiency analysis to study how diabetes care and individuals with diabetes co-work to produce capabilities and well-controlled risk factors. An intermediate model turned judgment of various aspects of support from diabetes care into patient abilities to manage diabetes and lifestyle factors, which in turn became capabilities in the form of wellbeing, and well-controlled risk factors, in a capability model.

The models were able to discriminate between most individuals (i.e. rank them), although a minor fraction came to comprise the frontier (fully efficient) and the models cannot discriminate between these. We saw logical associations between the output production and efficiency in both the intermediate and the capability models (Fig. 4), showing than efficient production is associated with beneficial output levels of PROM and risk factors.

Although the risk factors are the strongest contributors to capability efficiency, wellbeing is relatively stronger in efficient (successful) individuals, and risk factors relatively stronger in inefficient (less successful) individuals. It appears like risk factors have potential to give support for basic quality of life, but PROM are needed to reach higher quality of life. We take this as support for PROM being an important complement to risk factors in diabetes. However, the pattern of shares in relation to efficiency is heterogeneous, and we take this as support for our idea in the outset that the importance of different aspects varies between individuals. It has been suggested that a segmentation of the patient population based on behavior, expectations and needs would improve how healthcare manages patients [30]. It seems our findings suggest this as well. Future work may be required to explore this further.

We studied subgroups by gender, age and diabetes duration. Efficiency appears to explain differences between age groups, but it does not appear to explain differences between gender or duration groups. The differences between subgroups may appear modest, e.g. the greatest ranges were narrow, 0.65–0.72 (intermediate) and 0.91–0.93 (capability) between age groups. But efficiency is measured relative to the front, by comparing every individual to its similar peers. Though the fronts themselves may differ between subgroups, our relative measure doesn’t. This is consistent with allowing individual sets of importance weights, and with relating an individual to its own capability space. So, subgroups may have different fronts, i.e. different segments of the front.

We examined production of the abilities to manage diabetes and lifestyle factors at the diabetes clinics with the highest and lowest efficiency, and at the clinics with the highest levels of inputs, and we studied output shortfall compared to a reference clinic having the highest inputs. The shortfall was mainly due to inefficiency, however if everyone at the clinic were to become efficient, there would still be some shortfall pertaining to lower input levels. To eliminate this last shortfall, additional inputs would be required. Given that the level of efficiency rather than the levels of inputs at a clinic explain output shortfall, one might suspect that either the clinics play no important role in this, or that their role is mainly mediated through their patients’ efficiency. A sensitivity analysis also including the clinics that had the lowest levels of each input gives the same results and conclusion (data not shown). These results could have policy implications. First, if different healthcare providers allocate different level of support to their patients, and this is reflected in patient health and capabilities, one might want to even out the allocation to accomplish better equality in health and capabilities. Next, one could try to make inefficient (less successful) individuals more efficient. An example is that the NDR regularly publishes data on risk factors in the diabetes population [31], e.g. in different age groups, and an individual can compare with peers and see whether there is room to improve. Our proposed method, applied in clinical practice, would provide a complement to this on the individual level. In a sense, this would prompt the individual to express more outputs, using whatever tools they might have at hand to improve their efficiency, regardless of the cause of inefficiency. Another approach would be trying to understand the underlying causes of inefficiency and attempting to resolve them. This could be on the individual level, or at the clinic level. A potential outcome might be to discover that causes of inefficiency are rather a matter of resource allocation, e.g. need for a certain type of support, due to e.g. low age (parents must support), dementia or mental health (next of kin could support, possibly society). In principle such factors could be included as inputs in the efficiency analysis, in which case reducing inefficiency turns into a question of allocating inputs. On the other hand, if inefficiency persists or cannot be related to any identifiable inputs, one could consider compensating inefficiency by allocating more resources (support).

A case study of best practice could lead to insights, and if explanatory factors are discovered, whether they are intervenable or not will guide further steps, finding and encouraging effective ways for healthcare and patients to co-produce good health and capability [32]. Whether interventions are effective could be studied using the efficiency measure and inefficiency-related output shortfall. Input-related output shortfall could be studied to investigate the effect of allocation new levels of inputs. One clinical finding of ours was that the group aged 36–50 suffered from lower inputs, lower efficiency and lower output in the intermediate model than the other age groups. It would seem this age group would be a suitable starting point for the approaches suggested above. Thus, our method may provide insights into the causes of poor quality of life, but it should also be incorporated into wider systematic work to develop diabetes care and to ease living with diabetes. Used in clinical day-to-day practice, it has the potential to demonstrate the contribution of PROM, PREM and risk factors to quality of life.

Our general aim was to develop a single measure of quality of life for individuals with type 1 diabetes, based on PROM, PREM and risk factors. Two important features of the measure were (i) to enable comparisons without ambiguity, and (ii) not require any specific set of weights of the different variables, nor require the same set of weights for everyone. It seems that the application of efficiency analysis using DEA successfully met our general aim. Furthermore, our specific aims were to demonstrate feasibility of the application of efficiency analysis, to use it to study needs and improvement potential in subgroups, and to outline steps to intervene on causes of poor quality of life. We found the approach feasible, and we made clinical findings of need for improvement, and outlined steps to take the efficiency analysis and these findings further. Thus, it appears these specific aims were met as well.

Traditional use of efficiency analysis is with production units that use input resources to produce goods and services. This differs somewhat from our application, however we judged that most of the assumptions necessary for applying efficiency analysis were met. One exception is the presence of noise. Our analysis challenges the DEA assumption that there is no noise in the data. For a first study, we judged that this weakness would be acceptable, however there are DEA approaches to handle noise and this could be further pursued in a future study. Alternatively, should we have used Stochastic Frontier Analysis instead? This method manages noise in the data. Perhaps, but if multi-output production is important, prices are difficult to define and behavioral assumptions like cost-minimization or profit-maximization are hard to justify, DEA may often be the optimal choice [11]. Further, we applied restrictions on the weights on inputs and outputs, to ensure none were considered completely unimportant. A disadvantage with using restrictions on the relative importance of inputs and outputs is that projections, i.e. the points on the frontier to which individuals are compared, may lie outside the production set spanned by the original observations [6]. However, we used a wide range of restrictions to study the impact of the choice of restrictions and found the results to be stable over this range. The restrictions are directly linked to how far outside the production set a projection can be made, and the stability indicates that imposing these restrictions did not have any severe effect on our analysis.

Our scales, being limited between 0 and 100, may appear a problem as DEA requires that the inputs have no restrictions. Our inputs are restricted only by how we measure them. The scales could be extended to allow input measurement over a wider range. Another aspect is that the individuals with diabetes cannot change or affect the PREM inputs themselves, i.e. the inputs may appear non-discretionary and to be treated as such in the DEA. But we view this in a wider context, that developing diabetes care can affect the PREM inputs (presumably an improvement of care is given a more beneficial judgment). So ideally, by reporting low PREM, and therefore being inefficient (less successful), the individuals will drive development of diabetes care and hence increase the inputs available. For this reason, we treated the PREM as ordinary inputs instead of non-discretionary inputs, even though they differ principally from pure resource quantity inputs (commodities). Rather, they represent the individuals’ revealed preferences for healthcare, by describing their experience of healthcare encounters. A third aspect on availability of inputs is the individual’s endowment of health capital. We have only addressed this on an abstract level in our conceptual model (see below). Perhaps this can be elaborated more in a future work.

DEA, like any analytic model, may be sensitive to the choice of inputs and outputs, which in essence defines what is measured by the model. For the capability model, we selected input and outputs among patient-reported values considered important by individuals with diabetes [21, 22], and well-known risk factors for diabetes comorbidities. We may well have omitted other important variables from our analysis, but we do have a set of variables identified as important, with a comparably broad scope. The present work is a first step, and additional variables could be included in future studies. However, some of our variables could play other roles than the ones we assumed. For instance, we assumed that freedom from worries and limitations are inputs in the capability model. Should they have been outputs instead? One could argue that they are capabilities, e.g. resilience against limitations (and hence outputs), as well as something which is a requirement for wellbeing (and hence inputs). However, we judged the latter was their dominating role, and we therefore used them as inputs. Furthermore, we used risk factors as outputs in the capability model. Risk factors at problematic levels would be signs of more severe illness, more poorly managed illness or harder-to-manage illness. However, in such a situation we consider the risk factor levels to be signals of these circumstances rather than being their cause. Hence, we prefer to use them as outputs. A more general aspect of our production models is that they are unknown, we do not know the nature of the production process. We may know exactly how to forge iron into nails - using iron, tools, energy - but we don’t know how to create general wellbeing. However, this should not be a unique situation. Besides, chances are that not the exact same production process is at work in every individual. Perhaps the different views of what aspects are important bear witness of this.

Furthermore, we assumed a conceptual model of what defines an individual’s mix of outputs. We considered the distance to the front as the individual’s improvement potential, however the conceptual model may impose limitations to feasible production, such that failure to identify an individual’s front properly might lead to using a reference point outside the capability space. Whether this is the case cannot be determined with our current data, but this could be investigated in a future study e.g. involving empirical data before and after an intervention.

In our analysis of clinics, our finding that output shortfall was mainly due to inefficiency might indicate that the observed variation in inputs is small, or that we have not fully captured the production processes. Negative correlation between inputs and efficiency would indicate the latter as well, i.e. a lack of correspondence between input and output, however none of the correlations were negative, thus not lending any strength to the apprehension that the production processes were poorly captured.

The analyses of inefficiency and causes for output shortfall were made without accounting for any budget restriction. Doing so is complicated by the fact that not all costs fall upon the individual with diabetes. The gap between expectations of services and the offered services might be judged with incomplete knowledge about the cost reasons for this gap. Output shortfall due to lower level of inputs might reflect an intended level of allocation of resources, although it may be perceived by the individual as a lack of resources. Furthermore, the production of outputs involves the individual, who has an inherent time restriction. Inefficient production of output might stem from reluctance to spend time on this rather than on other more preferred activities.

We argue that the capability efficiency is a measure of quality of life, because it measures relative achievement in terms of patient capabilities (PROM) and health in the form of well-controlled risk factors. This should be consistent with measuring quality of life. It does not capture all aspects of quality of life, but the PROM and PREM scales capture several aspects considered important by individuals with diabetes [21, 22]. Furthermore, the risk factors used, HbA1c, SBP and LDL, are prognostic factors for cardiovascular and diabetes complications [33], hence important aspects of health in individuals with diabetes. We lack the data for comparing our measure to others, e.g. the EQ-5D. But this could be made in a future study. Although it remains to be seen, we anticipate that our measure can be developed into a complement rather than a competitor. Our DEA front should represent a maximum level of quality of life, whereas the capability efficiency is measured relative to the front, thus representing relative achievement. Other measures such as the EQ-5D Index appear to measure absolute levels of quality of life. How the front and the efficiencies relate to absolute measurements need to be explored further.

The present work was carried out using data from individuals with type 1 diabetes. If used for resource allocation, it is an obvious limitation that considerations of allocation can only be made within this condition. Our work can be extended to cover type 2 diabetes as well, but our PROM and PREM scales and risk factors are diabetes specific, so extension beyond diabetes appears infeasible i.e. across disease areas, and indeed outside healthcare. However, our approach should be useful in diabetes. It could also be extended to other chronic diseases in future studies.

## Conclusions

We used efficiency analysis of patient-reported outcomes measures, patient-reported experience measures and comorbidity risk factors to estimate quality of life with a broad focus on the individual. In the production of the abilities of managing diabetes and lifestyle factors, shortfall was to some extent due to lacking availability of inputs such as patient-judged support and access, but mainly due to inefficiency whose cause remain to be discovered. The approach appears useful for evaluating the situation of individuals with type 1 diabetes, in a way that allows ranking and comparisons, and that allows individuals to express different views on which aspects are important to them. This was also indicated empirically in the aspects’ contributions to the quality of life measure (i.e. share of the efficiency). E.g. for some individuals, well-controlled risk factors seem to drive basic quality of life, but wellbeing is needed for higher levels of quality of life. Our approach can be used for policy regarding interventions on inefficiency as well as healthcare resource allocation, although currently limited to type 1 diabetes.

## Supplementary information

**Additional file 1.** Supplementary material.

## Data Availability

The data underlying this study are confidential. For inquiries, please contact the corresponding author or the National Diabetes Register, Centre of Registers Västra Götaland, SE-413 45 Gothenburg, Sweden.

## Abbreviations

CM: Capability model
DEA: Data envelopment analysis
EQ-5D: EuroQol questionnaire
HbA1c: Glycated hemoglobin level
IM: Intermediate model
IRT: Item response theory
LDL: Low-density lipoprotein cholesterol
NDR: The Swedish National Diabetes Register
PREM: Patient-reported experience measures
PROM: Patient-reported outcomes measures
SBP: Systolic blood pressure
WTP: Willingness to pay
