# Supplementary material for: Quality of life in chronic conditions using patient-reported measures and biomarkers: a DEA analysis in type 1 diabetes
Source: Health Econ Rev. 2019 Nov 7;9:31. doi: 10.1186/s13561-019-0248-4 (PMC6836539; doi:10.1186/s13561-019-0248-4)

Additional file 1: Supplementary material.

Table S1: Correlations (Spearman) between risk factors and IRT Scores.

	**HbA1c**	**SBP**	**LDL**	**GenW**	**MoE**	**FreE**	**ManD**	**DiEx**	**NLD**	**NLBS**	**SuO**	**SuDC**	**AcDC**	**CoDC**	**MDMT**
HbA1c	1.00*														
SBP	0.05	1.00*													
LDL	0.09*	0.00	1.00*												
GenW General Wellbeing	-0.11*	0.07	-0.01	1.00*											
MoE Mood and energy	-0.12*	0.12*	-0.04	0.56*	1.00*										
FreW Free of worries	-0.14*	0.08	-0.04	0.39*	0.60*	1.00*									
ManD Manage your diabetes	-0.23*	0.04	-0.07	0.41*	0.60*	0.55*	1.00*								
DiEx Diet and exercise	-0.20*	0.08	-0.03	0.37*	0.42*	0.34*	0.47*	1.00*							
NLD Not limited by diabetes	-0.06	0.02	-0.04	0.31*	0.51*	0.46*	0.44*	0.28*	1.00*						
NLBS Not limited by blood sugar	-0.13*	0.03	-0.03	0.35*	0.48*	0.51*	0.43*	0.27*	0.58*	1.00*					
SuO Support from others	-0.08	0.02	-0.03	0.29*	0.36*	0.30*	0.42*	0.31*	0.31*	0.29*	1.00*				
SuDC Support from diabetes care	-0.05	0.02	-0.04	0.31*	0.37*	0.28*	0.37*	0.23*	0.31*	0.26*	0.34*	1.00*			
AcDC Access to diabetes care	-0.05	0.04	-0.04	0.29*	0.36*	0.32*	0.37*	0.25*	0.31*	0.29*	0.35*	0.62*	1.00*		
CoDC Continuity in diabetes care	0.06	-0.03	-0.02	0.08	0.07	0.07	0.12*	0.09	0.13*	0.07	0.15*	0.33*	0.33*	1.00*	
MDMT Medical devices and medical treatment	-0.00	0.05	-0.08	0.27*	0.40*	0.30*	0.39*	0.27*	0.32*	0.23*	0.32*	0.48*	0.42*	0.22*	1.00*

Notes: HbA1c = glycated hemoglobin level, SBP = Systolic Blood Pressure, LDL = Low-Density Lipoprotein cholesterol level. * = $p<0.001$.

Sensitivity analyses with regards to K.

Table S2: Efficiency in sensitivity analyses with regards to restriction K (proportion on the front, mean efficiency and 95% confidence interval), input and output weights for the intermediate and capability models.

(a) Intermediate model

		Input weights*				Efficiency	Output weights*	
**K**	**On the front**	**SuDC**	**AcDC**	**CoDC**	**MDMT**	**Mean (95% CI)**	**ManD**	**DiEx**
5[a]	3.8%	0.18	0.26	0.36	0.20	0.68 (0.35; 1.00)	0.65	0.35
10	4.1%	0.17	0.24	0.37	0.22	0.70 (0.36, 1.00)	0.68	0.32
20	4.2%	0.16	0.23	0.36	0.25	0.70 (0.37, 1.00)	0.69	0.31

(b) Capability Model

		Input weights*					Efficiency	Output weights*				
**K**	**On the front**	**FreW**	**ManD**	**DiEx**	**NLD**	**NLBS**	**Mean (95% CI)**	**GenW**	**MoE**	**HbA1c'**	**SBP'**	**LDL'**
5[a]	8.5%	0.16	0.27	0.28	0.10	0.19	0.92 (0.80, 1.00)	0.12	0.14	0.26	0.24	0.25
10	12.1%	0.16	0.28	0.27	0.12	0.17	0.93 (0.82, 1.00)	0.10	0.16	0.25	0.24	0.25
20	14.2%	0.16	0.26	0.27	0.12	0.18	0.94 (0.83, 1.00)	0.09	0.17	0.25	0.23	0.25

Notes: * Mean weights, normed to unit sum. ' = transformed. CI=confidence interval. [a] The main analysis used K=5. SuDC = Support from diabetes care, AcDC = Access to diabetes care, CoDC = Continuity in diabetes care, MDMT = Medical devices and medical treatment, ManD = Manage your diabetes, DiEx = Diet and exercise, FreW = Free of worries, NLD = Not limited by diabetes, NLBS = Not limited by blood sugar, GenW = General wellbeing, MoE = Mood and energy, HbA1c = glycated hemoglobin level, SBP = Systolic Blood Pressure, LDL = Low-Density Lipoprotein cholesterol.

Figure S1: Efficiency in the intermediate and capability models, in sensitivity analyses with regards to restriction K.

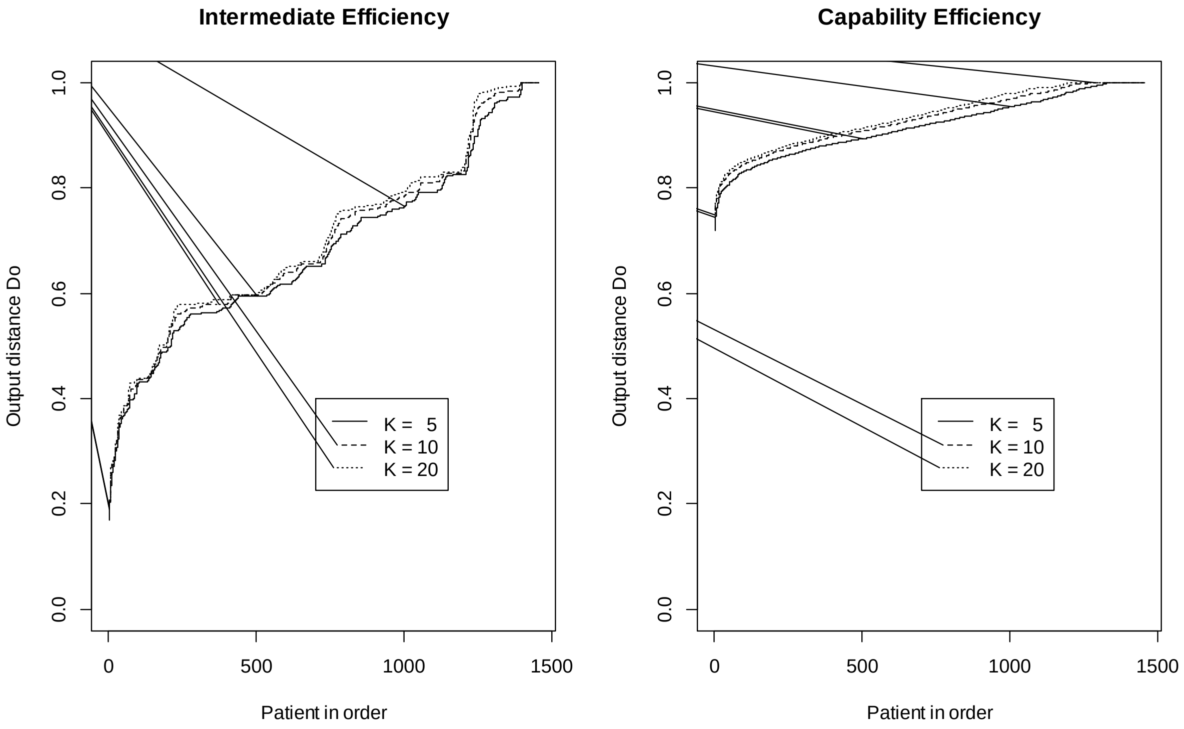

Supplement: Supplementary file 1 — Additional file 1. Supplementary material. [file 13561_2019_248_MOESM1_ESM.pdf]
